# Pre-Clinical Tests of an Integrated CMOS Biomolecular Sensor for Cardiac Diseases Diagnosis

**DOI:** 10.3390/s17122733

**Published:** 2017-11-26

**Authors:** Jen-Kuang Lee, I-Shun Wang, Chi-Hsien Huang, Yih-Fan Chen, Nien-Tsu Huang, Chih-Ting Lin

**Affiliations:** 1 Graduate Institute of Biomedical Electronics and Bioinformatics, National Taiwan University, Taipei 10617, Taiwan; b85401104@gmail.com (J.-K.L.); nthuang@ntu.edu.tw (N.-T.H.); 2 Division of Cardiology, Department of Internal Medicine, National Taiwan University Hospital, Taipei 10048, Taiwan; 3 Telehealth Center, National Taiwan University Hospital, Taipei 10048, Taiwan; 4 Department of Laboratory Medicine, National Taiwan University Hospital, Taipei 10048, Taiwan; 5 Graduate Institute of Electronics Engineering, National Taiwan University, Taipei 10617, Taiwan; i.shunwang@gmail.com; 6 Department of Materials Engineering, Ming Chi University of Technology, New Taipei 24301, Taiwan; chhuang@mail.mcut.edu.tw; 7 Insisute of Biophotonics, National Yang-Ming University, Taipei 11221, Taiwan; chenyf@ym.edu.tw

**Keywords:** CMOS biosensor, field-effect biosensor, heart disease, troponin-I, *N*-terminal prohormone brain natriuretic peptide, interleukin-6

## Abstract

Coronary artery disease and its related complications pose great threats to human health. In this work, we aim to clinically evaluate a CMOS field-effect biomolecular sensor for cardiac biomarkers, cardiac-specific troponin-I (cTnI), *N*-terminal prohormone brain natriuretic peptide (NT-proBNP), and interleukin-6 (IL-6). The CMOS biosensor is implemented via a standard commercialized 0.35 μm CMOS process. To validate the sensing characteristics, in buffer conditions, the developed CMOS biosensor has identified the detection limits of IL-6, cTnI, and NT-proBNP as being 45 pM, 32 pM, and 32 pM, respectively. In clinical serum conditions, furthermore, the developed CMOS biosensor performs a good correlation with an enzyme-linked immuno-sorbent assay (ELISA) obtained from a hospital central laboratory. Based on this work, the CMOS field-effect biosensor poses good potential for accomplishing the needs of a point-of-care testing (POCT) system for heart disease diagnosis.

## 1. Introduction

Coronary artery disease (CAD) remains the most important and threatened disease in both developed and developing countries and causes thousands of deaths every year [1]. Although CAD was formerly considered a lipid disease, it is now believed that the development of CAD is mainly from chronic inflammation [2]. If CAD is not diagnosed and treated well, myocardium loss, such as myocardial infarction, leads to impaired cardiac contractility and congestive heart failure. Therefore, three stages, i.e., inflammation, myocardial infarction, and heart failure, are important and unique phenomena in discussing the issue of CAD. There are numerous useful molecular biomarkers to help clinical health professionals to identify each stage of CAD [3,4]. For instance, in recent studies, evidences show that chronic inflammation may lead to coronary artery atherosclerosis, which is mediated by various inflammatory cytokines [4,5,6]. Interleukin-6 (IL-6) is one of the most important cytokines and is widely used in the cardiovascular laboratories for cardiovascular disease evaluation [7,8]. Furthermore, the inflammation may lead to atheroma plaque rupture and myocardial infarction with elevated cardiac biomarker, cardiac-specific troponin-I (cTnI). This biomarker specifically refers to cardiac cell death, and guidelines suggest it as the first choice of biomarker for myocardial infarction [9,10]. Finally, cardiac dysfunction developed with myocardium necrosis, i.e., congestive heart failure. At this stage, *N*-terminal pro–B-type NP (NT-proBNP) has been proposed as best biomarker in the diagnosis of congestive heart failure [11,12]. It could also be used to predict the clinical outcome, e.g., re-admission or death. Each biomarker mentioned above has its unique role in the diagnosis of heart disease. In fact, these three scenarios, inflammation, myocardial infarction, and congestive heart failure, often occur in concert. Therefore, measuring multi-biomarkers simultaneously is helpful for health professionals to diagnose disease and treat patients in time [13]. To achieve this, it is necessary to develop a platform with multi-biomarker and easy-to-check capabilities for clinical professionals.

To address above unmet needs of biomolecular diagnosis in clinical applications, several micro sensing technologies have been proposed for point-of-care (POC) applications to detect cardiac biomarkers. For instance, complementary-metal-oxide-semiconductor (CMOS) compatible biosensors [14], surface plasmon resonance (SPR) [15], quartz crystal microbalance (QCM) [16], and electrochemical impedance spectroscopy (EIS) [17] have been demonstrated to have label-free, real-time, and ultra-sensitive detection in designed buffers. Among these developed biomarker micro-sensing technologies, CMOS compatible biosensing technology is intriguing because of its ease of integration with wearable devices or mobile platforms [18]. However, most previously demonstrated CMOS-based biomarker sensing technologies have not been based on clinical samples, e.g., human serum or plasma. To pave the way toward POC diagnosis for cardiac disease, in this work, CMOS field-effect biosensing technology is employed for cardiac disease diagnosis with multiple biomarkers in serum. Compared with another CMOS-compatible micro-cantilever biosensing technology, CMOS field-effect biosensing technology has advantages of fabrication and integration with interface circuits and microfluidity [19].

To achieve the aforementioned goal, we designed a poly-silicon wire-form based biosensor device. The designed device was implemented via a standard commercialized 0.35 μm CMOS process. To show cardiac disease diagnosis, in this work, three cardiac biomarkers, IL-6, cTnI, and NT-proBNP, were chosen as our detection targets. Different kinds of samples, including buffers and human serums (IRB 201505009DINC), were used to validate and examine the developed CMOS based sensor system-on-chip (SSoC) technology. This work preliminarily demonstrates an point-of care testing (POCT) device that could evaluate the patient’s sophisticated cardiovascular status quickly and correctly.

## 2. Materials and Methods

### 2.1. Materials

Monoclonal anti-cTnI, cTnI protein, polyclonal anti-IL-6 and IL-6 protein were all purchased from AbCam, Inc. (Cambridge, UK). Monoclonal anti-NT-proBNP and NT-proBNP protein were purchased from Meridian Life Science, Inc. (Memphis, TN, USA). At the same time, chemicals used in this work, phosphate buffer saline (PBS), 3-aminopropyltriethoxysilane (≥98%, APTES), glutaraldehyde (GA, 50% aqueous solution), and bovine serum albumin (BSA), were all purchased from Sigma-Aldrich (St. Louis, MO, USA). Diluted PBS, 0.01× PBS with different pH values, was prepared by diluting 1× PBS (10 mM phosphate, pH 7.4) with ultrapure water for measurement. The ionic strength of 0.01× PBS is 1.8 mM, and the Debye length of the PBS buffer is around 7.3 nm [20]. All other chemicals used in this study were reagent grade.

### 2.2. Design of CMOS Field-Effect Sensing Device

**Figure 1:**
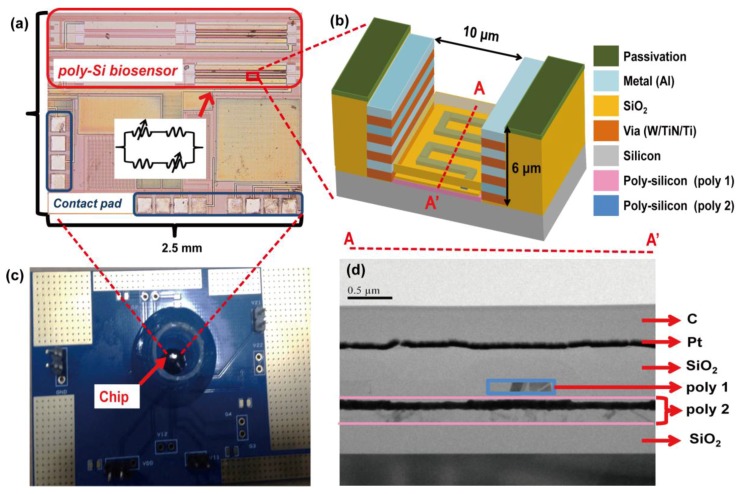
(**a**) A chip photo of the fabricated CMOS poly-silicon biosensor in the arrangement of Whetstone bridge architecture; (**b**) a schematic of the cross section of designed CMOS poly-silicon biosensor; (**c**) a bio-measurement module with CMOS poly-silicon biosensor chip wire-bonded within a plastic reservoir; (**d**) a TEM picture of the cross section of fabricated CMOS poly-silicon biosensor.

To accomplish the purpose of low-cost and mass production, poly-Si wire-form biosensors were manufactured by a 0.35 μm two-poly-four-metal (2P4M) commercially available CMOS fabrication technology. The detail poly-Si biosensor design was described in our previous work [18]. In this 2P4M technology, there are two poly-silicon layers and four metal layers. According to previous research reports [21], a low doping concentration semiconductor, compared to a high doping concentration semiconductor, has improved biosensing sensitivity. As a consequence, the second layer of poly-silicon (Poly 2) was selected and designed as the poly-Si biosensor due to the lower N-type doping level compared to the first layer of poly-silicon (Poly 1). To establish a measurable sensor arrangement, a Whetstone bridge architecture with four poly-silicon sensing devices was employed in order to reduce the noise from the environment and variations during the process, as shown in Figure 1a. Two of the poly-silicon sensing devices were exposed to perform biosensoring function after post-etching processes. The other two poly-silicon sensing devices were covered by a thick oxide layer to function as references. The biosensing area, which is the region of the two exposed poly-silicon sensors, was defined by the mask of the top-metal layer, i.e., the PAD layer, to remove the Si_3_N_4_ passivation layer. To expose the two designed poly-silicon sensors, after standard CMOS processes, a post process with reactive ion etching (RIE) and buffered hydrofluoric acid (BHF) wet etching were used, as shown in Figure 1b. Finally, as shown in Figure 1c, the fabricated biosensor chip was bonded on a printed-circuit-board (PCB) with a plastic reservoir. The reservoir was used for chip incubations of buffers and clinical samples in biomolecular detection experiments. It should be noted that there is an oxide layer around 300 nm thick on top of the poly-silicon sensor and that the oxide thickness was controlled by the wet etching. The transmission electron microscope (TEM) cross section of the fabricated poly-Si biosensor is shown in Figure 1d.

### 2.3. Surface Modification and Immobilization

To achieve specificity of biomarker detection, a surface functionalized process was introduced to functionalize the exposed poly-silicon biosensor to immobilize the specific antibody for the target protein. In brief, the poly-silicon exposed region was treated in 2% absolute ethanol solution of APTES for 1 h, followed by rinsing thoroughly with ethanol for salinization. To change the amine-terminated surface into an aldehyde-terminated surface, the 2.5% glutaraldehyde (GA) solution was used for 1 h in room temperature. Finally, antibodies for each biomarker were placed drop-wise onto the device. The entire module was stored in a 4 °C environment overnight. Before measurement, bovine serum albumin (BSA) with phosphate buffered saline (PBS) buffer solution was applied on the chip surface to block the surface. Then, the chip was ready for measurements after it was rinsed with PBS buffer.

### 2.4. Measurement Methods

To obtain the output response of fabricated poly-silicon biosensor, a picoammeter (Keithley 6485) was used. The experimental protocol can be briefly described as follows: (a) inject diluted PBS (0.01×) buffer solution for 5 min to obtain an initial reference base line; (b) inject a different concentration of the testing target biomarker (in PBS or human serum) and incubate for 5 min; (c) rinse the chip by diluted PBS (0.01×) three times to remove un-bound biomarkers; (d) measure the output voltage from the chip in a diluted PBS (0.01×) environment, i.e., the measurement buffer. In this work, all buffers and samples were 200 μL in volume in each pipetting procedure. For each experimental condition, it should be noted that the experimental results were extracted from 3 independent chips because of the limited number of chips. 

To extract an effective comparison between different chips, a normalized detection response (NDR) was defined as

(1) $$\frac{\Delta V}{V_{1}}=\left|\frac{\left(V_{S}-V_{0}\right)}{1.5V-V_{0}}\right|$$

where *V*_0_ is the output voltage measured from the initial background level in the diluted PBS buffer, and *V_S_* is the output voltage detected in the measurement buffer after the biomolecular binding process. The reference voltage in on-chip amplifier is 1.5 V.

## 3. Results

### 3.1. Measurement of Biomarkers in Buffer Solutions

**Figure 2:**
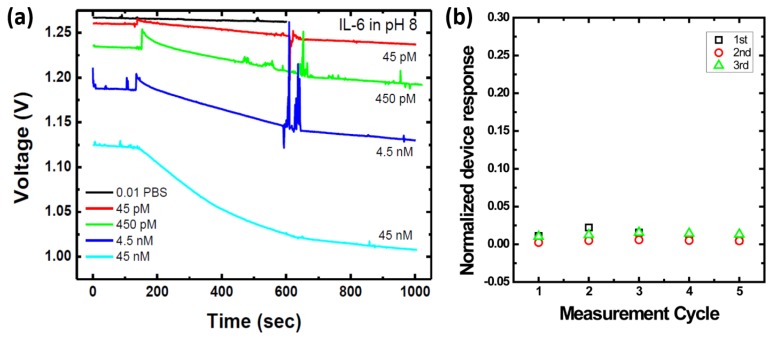
(**a**) A real-time experimental data of IL-6 measurement in PBS buffer solutions; (**b**) the experimental evaluation of base-line drift and noise of the sensor devices (*n* = 3). The sensing response of each chip was recorded five times, i.e., the buffer was washed and data were recorded. The result shows that NDR obtained in Figure 2a is attributed to specific binding of biomolecules.

In the first step, the sensing characteristics of the developed poly-silicon biosensors were examined by different biomarkers in buffer solutions. Although all three biomarkers, IL-6, cTnI, and NT-proBNP, have been examined, the experimental temporal response of IL-6 results are shown in Figure 2a to elucidate the examination process of the developed devices. It is clear that the sensor output voltage decreased as IL-6 concentration increased. It should be noted that the pH value of the measurement buffer used in the IL-6 experiments was 8.0. This is because the isoelectric point (pI) value affects the biomolecules’ net charge, which is the main factor in the field-effect sensing mechanism. The pI values of the three target biomarkers are different from each other. Based on previous reports, the pI values of IL-6, cTnI, and NT-proBNP are roughly 6.7, 5.2–5.4, and 6.3, respectively [22,23]. As a consequence, the pH value of the measurement buffer was 8.0, according to IL-6 measurements. At the same time, the pH value of the measurement buffer remained 7.4, according to both cTnI and NT-proBNP measurements.

At the same time, drifting and noise effects of the developed device was also evaluated in a lump-sum manner. To perform this evaluation, three sensor chips were used and the sensor surfaces were immobilized with anti-cTnI, i.e., cTnI antibody, by the surface modification protocol mentioned above. Then, the measurement buffer, i.e., diluted PBS buffer (0.01×), was introduced into the plastic reservoir on the sensor chip and the sensor response was recorded. These steps were repeated five times, e.g., five measurement cycles, shown in Figure 2b. Based on the evaluated results, the maximum NDR caused by drifting and noise effects was around 0.020, which is nearly one order less than the NDR measured from biomarker-detection experiments.

**Figure 3:**
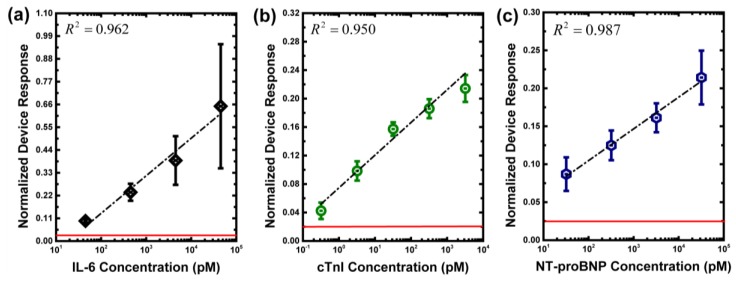
Experimental results of three biomarkers in PBS buffers. (**a**) IL-6; (**b**) cTnI; (**c**) NT-proBNP. The red line marks the noise level.

To examine functionalities of the developed CMOS poly-silicon field-effect biosensors, the sensor responses to the three biomarkers in PBS buffer environments were demonstrated. Figure 3 shows the experimental results of different biomarkers in PBS buffer conditions (*n* = 3). The detection limits of IL-6, cTnI, and NT-proBNP were 45 pM, 3.2 pM, and 32 pM, respectively. It can be noted that the sensor response variation of IL-6 at high concentration was much larger than that of cTnI and NT-proBNP. This might be because the pH level of the measurement buffer in IL-6 was adjusted to 8. At extremely high concentrations of binding complexes on the chip surface, as a consequence, different pH environments might lead to biomarker desorption from binding complexes on the chip surface [24]. In our experiments, the maximum drifting and noise level of IL-6, cTnI and NT-proBNP were 0.020, 0.020 and 0.025, respectively. To show this system’s noise level, in Figure 3, the red line marks the maximum drift and noise level. Figure 3 also shows that the coefficients of determination (R squared) values for IL-6, cTnI, and NT-proBNP were 0.962, 0.950, and 0.987, respectively.

### 3.2. Measurement of Biomarkers in Human Serums

After the developed devices were validated for these three cardiovascular disease biomarkers in buffer conditions, clinical human serum extracted from heart disease subjects were examined by the developed devices. This study was approved by the ethical committee of the National Taiwan University Hospital (IRB 201505009DINC), and all subjects provided written informed consent. In this study, the serum extracted from healthy people was used as a control sample in this study. The symptoms of subjects can be described as follows:

- Subject 1: healthy subject.
- Subject 2: subject with coronary artery disease; one-vessel disease; with medication control.
- Subject 3: subject with coronary artery disease; three-vessel disease; with medication control.
- Subject 4: subject with myocardial infarction; no shock; Killip Classification I: No evidence of heart failure.
- Subject 5: subject with myocardial infarction; no shock; Killip Classification II: Findings of mild to moderate heart failure and elevated jugular venous pressure.
- Subject 6: subject with congestive heart failure; outpatient clinic; NYHA functional class II: Slight limitation of physical activity. Comfortable at rest. Ordinary physical activity results in fatigue, palpitation, and dyspnea.
- Subject 7: subject with congestive heart failure; ICU admission; needs heart transplant; NYHA functional class IV: Unable to carry on any physical activity without discomfort. Symptoms of heart failure at rest. If any physical activity is undertaken, discomfort increases.
- Subject 8: subject with coronary artery disease; two-vessel disease; with medication control; admission due to myocardial infarction; shock with extracorporeal membrane oxygenation (ECMO); current congestive heart failure; ICU admission; Killip Classification IV: Cardiogenic shock defined as systolic blood pressure <90 and signs of hypoperfusion such as oliguria, cyanosis, and sweating.

**Figure 4:**
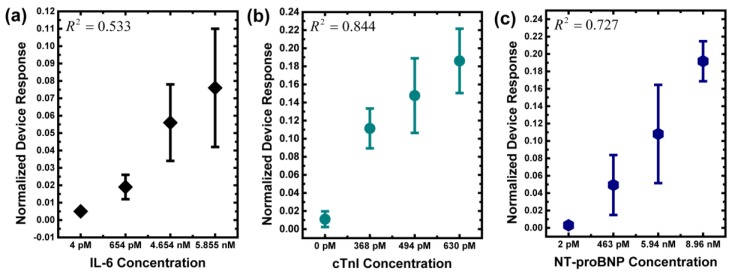
Experimental results of the three biomarkers in human serums. (**a**) IL-6; (**b**) cTnI; (**c**) NT-proBNP. In these plots, the x-axis represents the result measured by ELISA, and the y-axis represents the result measured by CMOS biosensors (*n* = 3). It should be noted that, to make the plot directly comparable to the clinical-sample ELISA read-out, the x-axis is not to scale.

Since each subject suffers from different symptoms, different biomarkers were examined, e.g., IL-6 was checked for coronary artery disease, cTnI was checked for myocardial infarction, and NT-proBNP was checked for congestive heart failure. To match the clinical examination, as a consequence, measurement with CMOS biomolecular sensing devices followed the examination of each subject. In other words, all human serum samples were examined with both the developed devices and the enzyme-linked immuno-sorbent assay (ELISA) of the central laboratory in National Taiwan University Hospital. The experimental results are shown in Table 1. To visualize the differences between ELISA and CMOS biosensor measurements, the experimental results of each biomarker are shown in Figure 4. Because there are other interference biomolecules in the human serum samples, the chip measurements in serum show larger variations than those in buffer [25,26]. Qualitatively speaking, it is clear that the developed CMOS biosensing device can be used to distinguish healthy people from patients with heart diseases. The R squared can be used to quantitatively evaluate our sensor performance versus the standard methods in clinical tests. The data show that the R squared values for IL-6, cTnI, and NT-proBNP are 0.533, 0.844, and 0.727, respectively.

## 4. Discussion

**Figure 5:**
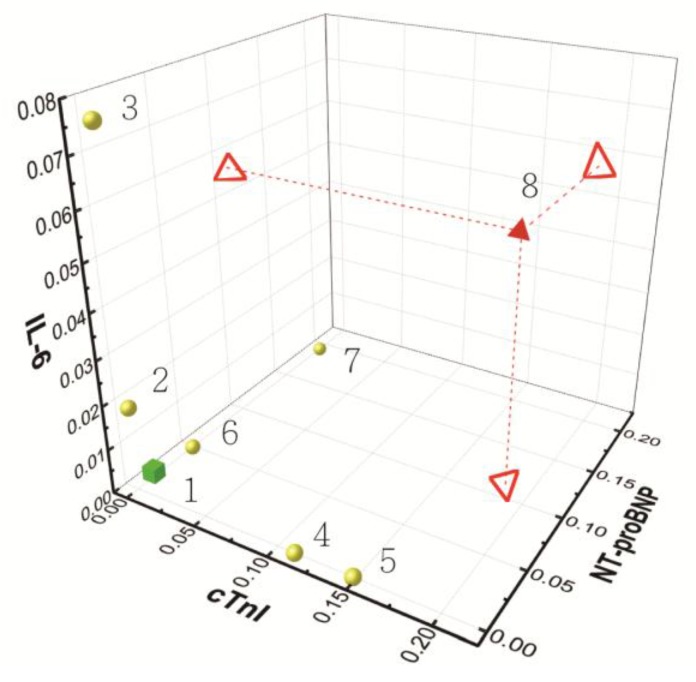
A presentation of the experimental results of biomarkers analysis in clinical human serums. The green cube represents the NDR of normal serum (Patient 1). The yellow spherical represents the NDR of a single heart disease (Patients 2–7). The red triangle cone represents the NDR of three heart diseases (Patient 8). The projected hollow triangles present the concentration in each biomarker.

Coronary artery disease and its related complications pose great threats to human health. In this work, we aimed to develop a CMOS-based biomolecular sensing device with good sensitivity for the detection of serum cardiac biomarkers, which may reflect the early changes of heart disease progression. At the first time, to our knowledge, this work clinically evaluates three major cardiac biomarkers, including IL-6, cTnI, and NT-proBNP, by CMOS-based semiconductor biosensor. These three biomarkers reflect the three important stages of atherosclerosis, i.e., coronary artery disease, myocardial infarction, and congestive heart failure, which comprise an intact picture for cardiovascular disease and its clinical outcome. IL-6 is a pro-inflammatory cytokine, which reflects the status of inflammation inside the human body. Generally, normal healthy people are under 461 pM, and patients with severe inflammation, e.g., major burns, are up to 23.1 nM [27,28,29]. cTnI is released into human blood once the myocardial cells are damaged. It is the best way of diagnosing myocardial infarction, which is one of the most dangerous situations doctors need to deal with [30]. The cut-off value is usually 2 pM, and it varies based on the assay being used [31]. The serum level is proportional to myocardium loss, which leads to cardiac necrosis and death. NT-proBNP increases when congestive heart failure takes place, and there is a dosage effect according to the NYHA class of CHF, I to IV. The serum level of healthy people is often below 347 pM, and doctors can easily evaluate the extent of CHF according to NT-proBNP level [32]. By combining these meaningful cardiac biomarker, we can differentiate the patient’s cardiac function in time effectively, whether in coronary artery inflammation, myocardium necrosis, or heart failure. Based on the experimental results in this work, the CMOS biosensor results show promising correlations with clinical scenarios, which are also validated with the same biomarkers examined by the central laboratory ELISA, as shown in Figure 5. In the three-biomarker analysis demonstrated here, physicians can have a good indication as to how patients with heart disease can be treated. In addition, a “cardiovascular panel” for individual monitoring with the developed CMOS biosensing devices is proposed.

In Figure 5, each unit of the axes is NDR, which is the readout of the developed biosensing chip. Nos. 1–8 corresponds to Subject Nos. 1–8 shown in Table 1. No. 1 is the healthy control, No. 2 and No. 3 are patients with coronary artery disease, No. 4 and No. 5 are patients with myocardial infarction, and No. 6 and No. 7 are patients with congestive heart failure. No. 8 is the patient with coronary artery disease, myocardial infarction, and congestive heart failure. After the serum biomarkers were measured with our integrated CMOS biomolecular sensor and projected onto the “IL-6, cTnI, NT-proBNP” 3D map, we could easily and quickly differentiate the cardiac stages of subjects. This panel test thus can help doctors interpret a patient’s cardiac condition and provide treatment within a short period of time. The green cube represents the NDR of normal serum (Patient 1). The yellow spherical represents the NDR of a single heart disease (Patients 2–7). The red triangle cone represents the NDR of three heart diseases (Patient 8). The projected hollow triangles present the concentration in each biomarker.

Though our works show promising results, there are several points to be improved. The first is the variation between different set of chips. Follow our developed post-process flow, the covering SiO_2_ etching process is manually controlled. For each set of chips, as a consequence, around four chips can be post-processed. Although quality control tests are used to minimize the post-process variation, the chip-to-chip variation suffers from a manual-controlled etching process. Furthermore, different processing chip sets suffer from the same problem. Based on our experimental results, variation within the same set, e.g., 3 chips, can be controlled within 0.02 NDR. However, the variation across different sets of chips could be much larger than that within the same set of chips. It is suspicious that this problem also contributes to the discrepancy between the experimental results of PBS buffers and those of human serums. This problem could be solved by supports from foundry services.

The second is the field-effect detection characteristic of different biomarkers. Different from the traditional fluorescent detection method, the field-effect detection utilizes the change of the surface charge induced by biomarker binding. It is clear that binding properties, such as polarity and orientation, of the antibody–antigen complex affect the performance of the developed CMOS biosensor. As a consequence, different biomarker detection methods require different optimized protocols to obtain optimal sensor performances, i.e., sensitivity and selectivity. Although this work demonstrates qualitative agreement between the CMOS biosensor and ELISA, unfortunately, protocol optimization is necessary. 

The final is the complexity of human serums. There are other biomolecules existing within serums. Most of them have net charges and a possibility of non-specific binding to the device surface. This leads to large variations in the measured data from the CMOS field-effect biosensors. This also results in problems of quantitative biomarker-concentration determinations in serums. Based on this work, therefore, it was concluded that a positive qualitative correlation between the developed CMOS field-effect biosensor and the ELISA can be achieved.

## 5. Conclusions

In this work, we developed a CMOS biomolecular sensor based on the field-effect detection mechanism. To examine the potential for clinical diagnosis of heart diseases, three major heart-disease biomarkers, cTnI, NT-proBNP, and IL-6, were employed in tests with both PBS buffer and clinical serum. In buffer conditions, the developed CMOS field-effect biosensor was identified to have appropriated detection ranges for all of three biomarkers. The detection limits of IL-6, cTnI, and NT-proBNP were 45 pM, 3.2 pM, and 32 pM, respectively. In clinical serum conditions, furthermore, the CMOS biosensor measurement data have a promising correlation with the ELISA measurement data obtained from the hospital central lab. Based on this work, several points regarding improvements for further development toward clinical applications were raised. Moreover, this is the first time that the potential of CMOS biosensors for a multiple-biomarker-based “cardiovascular panel” in clinical tests has been demonstrated.

## Figures and Tables

**Table 1:** The experimental results of IL-6, cTnI, and NT-proBNP in subject serums. It should be noted that the measurement units of ELISA have been transferred to molar concentration.

Sub. No.	ELISA (pM)			CMOS Biosensor (NDR)			Note (Symptoms)
	IL-6	cTnI	NT-proBNP	IL-6	cTnI	NT-proBNP	
1	4 ± 1.8	0 ± 0.0	2 ± 1.0	0.005 ± 0.001	0.011 ± 0.009	0.003 ± 0.001	Healthy people
2	654 ± 3.8	-	-	0.019 ± 0.007	-	-	coronary artery disease
3	5855 ± 5.9	-	-	0.076 ± 0.034	-	-	
4	-	368 ± 0.6	-	-	0.111 ± 0.022	-	myocardial infarction
5	-	494 ± 1.1	-	-	0.148 ± 0.041	-	
6	-	-	463 ± 0.2	-	-	0.049 ± 0.034	congestive heart failure
7	-	-	8960 ± 24.4	-	-	0.192 ± 0.023	
8	4654 ± 5.9	630 ± 6.4	5940 ± 24.6	0.056 ± 0.022	0.186 ± 0.036	0.108 ± 0.057	coronary artery disease & congestive heart failure & myocardial infarction
